# Association of different types of physical activity with sarcopenia and its parameters in peritoneal dialysis patients: an isotemporal substitution analysis

**DOI:** 10.3389/fnut.2026.1780207

**Published:** 2026-02-25

**Authors:** Hongyan Li, Yuxin Xiong, Xiaotian Zhang, Yuanhua Zheng, Li Wei, Ruixue Bi, Wei Luo, Xin Sun, Xinyue Hou, Gaoleer Yang, Yaqing Zhang

**Affiliations:** 1 School of Nursing, Jiangxi Medical College, Nanchang University, Nanchang, Jiangxi, China; 2 School of Nursing, Shanghai Jiao Tong University School of Medicine, Shanghai, China; 3 Peritoneal Dialysis Center, The First Affiliated Hospital of Nanchang University, Nanchang, Jiangxi, China; 4 School of Nursing, College of Health and Education, Murdoch University, Murdoch, WA, Australia; 5 Jiangxi Province Key Laboratory of Aging and Disease, Nanchang, Jiangxi, China; 6 Department of Nursing, Jiangxi Provincial People's Hospital, Nanchang, Jiangxi, China

**Keywords:** isotemporal substitution model, peritoneal dialysis, physical activity, sarcopenia, skeletal muscle

## Abstract

**Aim:**

The impact of different exercise modality on sarcopenia in peritoneal dialysis patients remain unclear. This study examined the association between physical activity and sarcopenia in peritoneal dialysis patients based on the Isotemporal Substitution Model and attempted to identify which specific exercise modality is more effective in patients with peritoneal dialysis.

**Methods:**

A cross-sectional analysis design was informed. From March 2023 to February 2024, patients undergoing peritoneal dialysis were recruited from the peritoneal dialysis centers of two general hospitals in China. Sarcopenia was diagnosed according to the 2019 criteria established by the Asian Working Group for Sarcopenia. Physical activity levels were evaluated using the Chinese Low Physical Activity Questionnaire. Isotemporal Substitution Model were employed to examine the association between sarcopenia in peritoneal dialysis patients and four types of physical activity (walking, light physical activity, moderate-to-vigorous physical activity, and daytime inactivity).

**Results:**

A total of 643 patients were recruited in the study for data analysis. The prevalence of sarcopenia in peritoneal dialysis patients was 12.6%. Among the participants, 69.67% engaged in light physical activity, 15.86% in moderate physical activity, and only 0.93% in vigorous physical activity. Substituting 10 min of daytime inactivity with 10 min of light physical activity was associated with a significant increase in skeletal muscle index (*β* = 0.007, 95% CI: 0.001–0.013, *p* < 0.05), a significant improvement in handgrip strength (*β* = 0.118, 95% CI: 0.031–0.149, *p* < 0.001), and a reduction in five-time sit-to-stand test (*β* = −0.045, 95% CI: −0.078 to −0.012, *p* < 0.01). Substituting 10 min of daytime inactivity with 10 min of walking was associated with a shorter five-time sit-to-stand test completion time (*β* = −0.077, 95% CI: −0.135 to −0.019, *p* < 0.05). However, substituting 10 min of daytime inactivity with light physical activity, moderate-to-vigorous physical activity, or walking showed no significant change in the occurrence of sarcopenia.

**Conclusion:**

Based on the Isotemporal Substitution Model, this study found that substituting daytime inactivity with light physical activity was significantly associated with three parameters of sarcopenia. Substituting daytime inactivity with walking was significantly associated with the five-time sit-to-stand test.

## 1 Introduction

With the increasing prevalence of chronic diseases such as diabetes, hypertension, and chronic kidney disease, the incidence of end-stage renal disease has been rising, making it a major global public health issue (1). Peritoneal dialysis is one of the renal replacement therapies for end-stage renal disease. It is widely adopted due to its simplicity for home-based treatment, minimal disruption to daily life, hemodynamic stability, slower loss of residual renal function, and lower treatment cost compared to hemodialysis (2).

Sarcopenia is one of the common complications in patients undergoing peritoneal dialysis and is a significant condition associated with high rates of disability, mortality, and morbidity (3). The global prevalence of sarcopenia among peritoneal dialysis patients ranges from 10 to 27% (4), while its prevalence in China is reported to be between 10.9 and 38.2% (5, 6). Studies indicate that peritoneal dialysis patients with sarcopenia experience reduced daily activity levels, increased risks of falls, disability, fatigue, depression, adverse cardiovascular events, hospitalization, and mortality (7). Dialysis treatment may lead to malnutrition, metabolic disorders, and accelerated muscle catabolism in peritoneal dialysis patients with sarcopenia, which in turn causes a decrease in muscle mass and strength.

The level of physical activity is closely associated with the occurrence of sarcopenia. Low levels of physical activity may further exacerbate the decline in motor function and muscle mass. The prevalence of insufficient physical activity and sedentary behavior ranges from 36 to 65.3% among patients receiving peritoneal dialysis (8, 9). A cohort study showed that the proportions of patients engaging in light-intensity, moderate-intensity, and vigorous-intensity physical activity were 31.6, 48.7, and 19.7%, respectively (10). The International Society for Peritoneal Dialysis emphasizes that muscle strength training, resistance exercise, and aerobic exercise play important roles in maintaining skeletal muscle mass, enhancing muscle strength, improving physical function, and promoting quality of life (11). for example, regular exercise training can significantly improve objective physical function indicators in dialysis patients, such as the 6-min walk test (6MWT) distance, sit-to-stand test results, and handgrip strength, effectively increase muscle mass and strength, and enhance cardiorespiratory fitness and overall endurance (12, 13). For peritoneal dialysis patients, structured exercise interventions, a type of planned, prescribed physical activities, particularly a combination of resistance and aerobic training, can effectively improve muscle condition (14, 15). However, existing exercise intervention studies have primarily focused on hemodialysis patients, with limited research targerting peritoneal dialysis patients with sarcopenia, and even fewer studies investigating appropriate exercise type and intensity for this population.

The Isotemporal Substitution Model is an analytic model to study the time-substitution effects of one activity for another (16). Due to time constraints, engaging in one type of physical activity will inevitably lead to the inability to participate in another type of physical activity or reduce the time available for other types of physical activity (16). Based on this premise, the model uses a multiple linear regression framework to establish a hypothetical analytical scenario (17), attempting to substitute one form of physical activity with another within the same time period, in order to observe the effects of different types of activity time on health outcomes.

The application of the Isotemporal Substitution Model has primarily focused on analyzing the substitution effects between physical activity and sedentary behavior. Studies have shown that substituting sedentary time with physical activity can significantly reduce the risk of obesity, cardiovascular disease, and metabolic syndrome (18, 19). In recent years, the Isotemporal Substitution Model has been further applied to investigate the potential effects of time reallocation among different physical activity behaviors on sarcopenia in older adults (20–23). Results indicate that substituting sedentary time with moderate-to-vigorous physical activity can significantly improve muscle mass and handgrip strength in the elderly, thereby reducing the risk of sarcopenia (21). Based on the Isotemporal Substitution Model, Chien recommended that older adults with sarcopenia should increase moderate-to-vigorous physical activity and reduce sedentary time to delay disease progression and improve quality of life (24). However, most peritoneal dialysis patients primarily engagein light physical activity and often lack the willingness or ability to participate in moderate-to-vigorous physical activity due to factors, such as fatigue, comorbidities or limited physical tolerance (25, 26). Despite this, few studies have examined the association between exercise type and muscle health in peritoneal dialysis patients with sarcopenia. Therefore, based on the Isotemporal Substitution Model, this study examined the potential effects of substituting daytime inactivity (sedentary/recumbent behavior) with walking, light physical activity or moderate-to-vigorous physical activity on sarcopenia among peritoneal dialysis patients. The aim was to identify the optimal exercise type and intensity, thereby providing an evidence basis for developing targeted exercise intervention strategies for peritoneal dialysis patients complicated with sarcopenia.

## 2 Materials and methods

### 2.1 Study design and participants

This study is a cross-sectional analysis designed to examine the associations between different types of physical activity and sarcopenia in peritoneal dialysis patients. Nurses assessed muscle mass, muscle strength, and physical function using the skeletal muscle index, handgrip strength, and five-time sit-to-stand test. Physical activity levels were evaluated with the Chinese Low Physical Activity Questionnaire (C-LoPAQ). The Isotemporal Substitution Model was used to examine the association between sarcopenia (and its three parameters: skeletal muscle index, handgrip strength, and five-time sit-to-stand test) and four types of physical activity (walking, light physical activity, moderate-to-vigorous physical activity, and daytime sedentary/recumbent behavior). All nurses who collected data received unified training; the measurement indicators were operated by the same researcher to ensure a consistent measurement method. Data were entered into the database by two people (in a double-entry manner). The study was approved by the ethics review committee of the study hospital [approval: (2023)CDYFYYLK(03–018)]. All participants provided written informed consent.

A convenience sampling method was employed. Between March 2023 and February 2024, patients were selected from the peritoneal dialysis centers of two large general hospitals in Nanchang, Jiangxi Province, China. According to the incidence of sarcopenia in peritoneal dialysis patients in China, referring to the research results of Shen et al. (25), which is approximately 13%, the calculated sample size was 643 cases. Based on the following inclusion criteria: aged ≥ 18 years; having received regular peritoneal dialysis for ≥1 month; being conscious and able to communicate verbally without impairment; capable of cooperating with handgrip strength and five-time sit-to-stand test measurements; eligible for body composition analysis; and having undergone peritoneal dialysis treatment with regular follow-up. Exclusion criteria were (i) patients concurrently receiving hemodialysis; (ii) patients with severe cardiac or hepatic insufficiency, respiratory failure, or active infection within the past month. Participants with incomplete follow-up data or incomplete questionnaire responses were also excluded.

### 2.2 Data collection

#### 2.2.1 Identification of sarcopenia

According to the diagnostic criteria for sarcopenia established by the Asian Working Group for Sarcopenia (AWGS) in 2019 (27), sarcopenia is defined as low muscle mass accompanied by low muscle strength and/or low physical function. Low muscle mass is determined by the skeletal muscle index. Low muscle strength was assessed by handgrip strength. Low physical function is evaluated using the five-time sit-to-stand test. The five-time sit-to-stand test correlates with knee extension strength and gait performance, and can well reflect lower limb function and physical performance (28). AWGS 2019 have proposed that the 5-time sit-to-stand test can serve as a surrogate marker for gait speed in the diagnosis of sarcopenia (27).

Skeletal muscle index: Peritoneal dialysis patients underwent bioelectrical impedance analysis after an overnight fast. Skeletal muscle mass was measured using a body composition analyzer (InBody 270, South Korea). Skeletal Muscle Index = Skeletal Muscle Mass (kg)/Height Squared (m^2^) (29).

Handgrip Strength: Handgrip strength was measured using an electronic dynamometer (CAMRY EH101, China). The maximum value obtained from three consecutive tests performed with the patient’s dominant hand was recorded.

Five-Time Sit-to-Stand Test: The time taken to complete five consecutive “stand-up and sit-down” movements as quickly as possible was recorded (28, 30).

#### 2.2.2 Physical activity assessment

The Chinese Low Physical Activity Questionnaire (C-LoPAQ), developed by Johansen et al. (31) and sinicized by Huang et al. (32), was adopted. We investigated the frequency and duration of three distinct types of walking (transportation walking, fitness walking, and leisure walking), the frequency and duration of activities with three different intensity levels (light, moderate, and high), as well as sedentary time and daytime napping time (recumbency) among patients over the past 30 days. These periods of time need to last for at least 10 min. Walking is classified as light physical activity, while sedentary time and daytime napping time (recumbency) are defined as daytime inactivity.

The calculation method for physical activity energy consumption was as follows: energy expenditure of different physical activity is measured in Metabolic Equivalent of Task (MET) units. The energy consumption per minute for various types of physical activity is defined as: Transportation walking, fitness walking, and leisure walking: 3.0 MET. Light Physical Activity: 2.0 MET. Moderate Physical Activity: 4.0 MET. Vigorous Physical Activity: 6.0 MET. Physical activity consumption was calculated based on MET values, activity type, weekly frequency, and duration. Physical activity consumption (MET-h/w) = MET × weekly frequency of each physical activity × daily activity time. The total physical activity consumption was the sum of the various types of physical activity consumption.

#### 2.2.3 Covariates

The study adjusted for gender, age, dialysis vintage, body mass index, comorbidity index, albumin, and C-reactive protein. The Age-adjusted Charlson Comorbidity Index was used to assess the comorbidity status of the patients. The Age-adjusted Charlson Comorbidity Index is calculated by summing the patient’s age-related score and Charlson Comorbidity Index score; a higher total indicates a greater severity of the patient’s condition (33).

### 2.3 Statistical analysis

This study utilized three distinct linear regression models to examine the association between sarcopenia (and its three parameters: skeletal muscle index, handgrip strength, and five-time sit-to-stand test) and four types of physical activity (walking, light physical activity, moderate-to-vigorous physical activity, and daytime sedentary/recumbent behavior). The models included a single-model, a partition-model, and an isotemporal substitution model. Both the International Physical Activity Questionnaire and the C-LoPAQ defined a continuous activity lasting at least 10 min as one unit of measurement. In line with other relevant studies (34), a 10-min duration was selected as the initial substitution unit. For analyzing the association between the four types of physical activity (walking, light physical activity, moderate-to-vigorous physical activity, and daytime sedentary/recumbent behavior) and the occurrence of sarcopenia in peritoneal dialysis patients, 12 logistic regression models were constructed, including four single-model analyses, four partition-model analyses, and four isotemporal substitution models. Similarly, for examining the association between physical activity levels and the three key parameters (skeletal muscle index, handgrip strength, and five-time sit-to-stand test), 12 linear regression models were established for each indicator, resulting in a total of 48 models. Normally distributed continuous variables were expressed as mean ± standard deviation (mean ± SD), while non-normally distributed variables were expressed as median (P_25_, P_75_). For continuous variables with a normal distribution, the independent samples t-test was used; for continuous variables with a skewed distribution, the Mann–Whitney U test was applied. For categorical data, the chi-square test or Fisher’s exact test was adopted. Statistical analyses were performed using IBM SPSS 26.0 software, with a two-tailed *p* < 0.05 considered statistically significant.

## 3 Results

### 3.1 Peritoneal dialysis patients’ basic characteristics

This study included 643 peritoneal dialysis patients, in which 81 (12.6%) were diagnosed with sarcopenia. The mean age of the total sample was 48.04 years, while the mean age of patients with sarcopenia was 53.27 years. Males accounted for 50.7% of the participants. The majority of peritoneal dialysis patients engaged in leisure walking (67.96%), followed by transport walking (13.37%), with fitness walking being the least common (11.98%). In terms of physical activity intensity, 69.67% participants performed light physical activity followed by moderate physical activity (15.86%), while vigorous physical activity accounted for 0.93% (see Supplementary Table S1).

### 3.2 The association between physical activity levels and sarcopenia incidence

#### 3.2.1 Single and partitioned models of physical activity levels and sarcopenia incidence

According to the single-model results, no statistically significant association was found between each individual physical activity, daytime inactivity, and the occurrence of sarcopenia (all *p* > 0.05). Results from the partition-model showed that for every 10-min increase in walking, light physical activity, or moderate-to-vigorous physical activity, however, there was no change in the incidence of sarcopenia (all *p* > 0.05) (see Supplementary Table S2).

#### 3.2.2 Effects of 10-min isotemporal substitution between different physical activities on sarcopenia incidence

The Isotemporal Substitution Model indicated that substituting 10 min of daytime inactivity with an equal duration of walking, light physical activity, or moderate-to-vigorous physical activity showed no statistically significant effect on the incidence of sarcopenia (all *p* > 0.05). Similarly, substituting 10 min of walking or light physical activity with an equal duration of moderate-to-vigorous physical activity also showed no statistically significant association with sarcopenia occurrence (all *p* > 0.05). No other substitutions yielded significant results (see Table 1).

**Table 1:** Effects of 10-min isotemporal substitution between different physical activities on sarcopenia incidence.

Category	Walking		LPA		MVPA		Daytime inactivity	
	*β*	*OR* (95%CI)	*β*	*OR* (95%CI)	*β*	*OR* (95%CI)	*β*	*OR* (95%CI)
①			0.046	1.047 (0.959, 1.143)	0.003	1.003 (0.867, 1.160)	0.063	1.065 (0.982, 1.155)
②	−0.046	0.955 (0.875, 1.043)			−0.043	0.958 (0.844, 1.087)	0.017	1.017 (0.979, 1.056)
③	−0.003	0.997 (0.862, 1.153)	0.043	1.044 (0.920, 1.185)			0.060	1.062 (0.941, 1.199)
④	−0.063	0.939 (0.866, 1.018)	−0.017	0.983 (0.947, 1.021)	−0.060	0.942 (0.834, 1.063)		

Adjusted for gender, age, dialysis vintage, body mass index, Age-adjusted Charlson Comorbidity Index, albumin, and C-reactive protein. LPA, light physical activity; MVPA, moderate-to-vigorous physical activity; ① Substituting walking; ② Substituting LPA; ③ Substituting MVPA; ④ Substituting daytime inactivity. ^*^p < 0.05; ^**^p < 0.01.

### 3.3 The association between physical activity levels and skeletal muscle index

#### 3.3.1 Single and partitioned models of physical activity levels and skeletal muscle index

After adjusting for gender, age, dialysis vintage, body mass index, Age-adjusted Charlson Comorbidity Index, albumin, and C-reactive protein, the variance inflation factors for all activity behaviors were less than 4 (maximum value: 1.195). This indicates that there was no multicollinearity among the activity variables, allowing for further analysis.

Both the single-model and the partition-model showed no statistically significant associations between any physical activity levels and skeletal muscle index (all *p* > 0.05) (see Supplementary Table S3).

#### 3.3.2 Effects of 10-min isotemporal substitution between different physical activities on skeletal muscle index

The Isotemporal Substitution Model indicated that substituting 10 min of daytime inactivity with an equal duration of light physical activity significantly increased muscle mass (*β* = 0.007, 95% CI: 0.001 ~ 0.013, *p* < 0.05). Conversely, substituting light physical activity with an equal duration of daytime inactivity resulted in a significant decrease in muscle mass of equal magnitude (*β* = −0.007, 95% CI: −0.013 ~ −0.001, *p* < 0.05). No other substitutions showed statistically significant effects (see Table 2).

**Table 2:** Effects of 10-min isotemporal substitution between physical activity components on skeletal muscle index.

Category	Walking	LPA	MVPA	Daytime inactivity
	*β* (95%CI)	*β* (95%CI)	*β* (95%CI)	*β* (95%CI)
①		−0.004 (−0.017, 0.008)	−0.002 (−0.022, 0.018)	−0.011 (−0.022, 0.001)
②	0.004 (−0.008, 0.017)		0.002 (−0.015, 0.019)	−0.007^*^ (−0.013, −0.001)
③	0.002 (−0.018, 0.022)	−0.002 (−0.019, 0.015)		−0.009 (−0.025, 0.007)
④	0.011 (0.001, 0.022)	0.007^*^ (0.001, 0.013)	0.009 (−0.007, 0.025)	

Adjusted for gender, age, dialysis vintage, body mass index, Age-adjusted Charlson Comorbidity Index, albumin, and C-reactive protein. LPA, light physical activity; MVPA, moderate-to-vigorous physical activity; ① Substituting walking; ② Substituting LPA; ③ Substituting MVPA; ④ Substituting daytime inactivity. ^*^P < 0.05; ^**^P < 0.01. Bold values in table indicate statistically significant results.

### 3.4 The association between physical activity levels and handgrip strength

#### 3.4.1 Single and partitioned models of physical activity levels and handgrip strength

After adjusting for gender, age, dialysis vintage, body mass index, Age-adjusted Charlson Comorbidity Index, albumin, and C-reactive protein, the variance inflation factors of different activity behaviors were all less than 4 (maximum value: 1.217). No multicollinearity was detected among these activity behaviors, which allowed for further analysis.

The single-model showed that 10 min of light physical activity had a significant positive effect on grip strength (*β* = 0.089, 95% CI: 0.033 ~ 0.145, *p* < 0.01); no significant differences were observed in other outcomes. Also, the partition-model indicated that every 10-min increase in light physical activity exerted a significant positive effect on grip strength (*β* = 0.092, 95%CI: 0.034 ~ 0.145, *p* < 0.01). However, no significant differences were found in other outcomes (see Supplementary Table S4).

#### 3.4.2 Effects of 10-min isotemporal substitution between different physical activities on handgrip strength

The Isotemporal Substitution Model indicated that substituting 10 min of daytime inactivity with an equal duration of light physical activity significantly increased grip strength (*β* = 0.118, 95%CI: 0.031 ~ 0.149, *p* < 0.001). Conversely, substituting light physical activity with an equal duration of daytime inactivity significantly decreased grip strength, and the magnitude of the decrease was equal to that of the increase (*β* = −0.090, 95%CI: −0.149 ~ −0.031, *p* < 0.001). No significant differences were noted in other substitution results (see Table 3).

**Table 3:** Effects of 10-min isotemporal substitution between different physical activities on handgrip strength.

Category	Walking	LPA	MVPA	Daytime inactivity
	*β*(95%CI)	*β*(95%CI)	*β*(95%CI)	*β*(95%CI)
①		0.018 (−0.096, 0.133)	0.066 (−0.247, 0.114)	−0.072 (−0.175, 0.031)
②	−0.018 (−0.133, 0.096)		−0.085 (−0.240, 0.071)	−0.090^**^ (−0.149, −0.031)
③	0.066 (−0.114, 0.247)	0.085 (−0.071, 0.240)		−0.005 (−0.152, 0.141)
④	0.072 (−0.031, 0.175)	0.090^**^ (0.031, 0.149)	0.005 (−0.141, 0.152)	

Adjusted for gender, age, dialysis vintage, body mass index, Age-adjusted Charlson Comorbidity Index, albumin, and C-reactive protein. LPA, light physical activity; MVPA, moderate-to-vigorous physical activity; ① Substituting walking; ② Substituting LPA; ③ Substituting MVPA; ④ Substituting daytime inactivity. ^*^P < 0.05; ^**^p < 0.01. Bold values in table indicate statistically significant results.

### 3.5 The association between physical activity levels and five-time sit-to-stand test

#### 3.5.1 Single and partitioned models of physical activity levels and five-time sit-to-stand test

After adjusting for gender, age, dialysis vintage, body mass index, Age-adjusted Charlson Comorbidity Index, albumin, and C-reactive protein in this study, the variance inflation factors of different activity behaviors were all less than 4 (maximum value: 1.217). No multicollinearity was observed among these activity behaviors, enabling further analysis.

The single-model showed that 10 min of walking time had a significant effect on reducing the five-time sit-to-stand test time (*β* = −0.066, 95% CI: −0.124 ~ −0.007, *p* < 0.05); 10 min of daytime inactivity had a significant effect on increasing five-time sit-to-stand test time (*β* = 0.027, 95%CI: 0.010 ~ 0.043, *p* < 0.01); no significant differences were found in other physical activity. According to the results of the partition-model, every 10-min increase of daytime inactivity exerted a significant positive effect on increasing five-time sit-to-stand test time (*β* = 0.022, 95%CI: 0.004 ~ 0.039, *p* < 0.05); no significant differences were observed in other results (see Supplementary Table S5).

#### 3.5.2 Effects of 10-min isotemporal substitution between different physical activities on five-time sit-to-stand test

The Isotemporal Substitution Model analysis showed that patients’ five-time sit-to-stand test time decreased significantly when 10 min of walking time substituted daytime inactivity (*β* = −0.077, 95%CI: −0.135 ~ −0.019, *p* < 0.05); and when 10 min of light physical activity substituted daytime inactivity (*β* = −0.045, 95%CI: −0.078 ~ −0.012, *p* < 0.01). Conversely, in reverse substitutions, five-time sit-to-stand test time increased significantly when 10 min of daytime inactivity replaced walking (*β* = 0.077, 95%CI: 0.019 ~ 0.135, *p* < 0.05) and light physical activity (*β* = 0.045, 95%CI: 0.012 ~ 0.078, *p* < 0.01). The magnitude of the increase mirrored that of the decrease. No significant differences were observed in other substitution results (see Table 4).

**Table 4:** Effects of 10-min isotemporal substitution between different physical activities on five-time sit-to-stand test.

Category	Walking	LPA	MVPA	Daytime inactivity
	*β*(95%CI)	*β*(95%CI)	*β*(95%CI)	*β*(95%CI)
①		0.032 (−0.033, 0.097)	0.059 (−0.043, 0.161)	0.077^*^ (0.019, 0.135)
②	−0.032 (−0.097, 0.033)		0.026 (−0.062, 0.114)	0.045^**^ (0.012, 0.078)
③	−0.059 (−0.161, 0.043)	−0.026 (−0.114, 0.062)		0.018 (−0.064, 0.101)
④	−0.077^*^ (−0.135, −0.019)	−0.045^**^ (−0.078, −0.012)	−0.018 (−0.101, 0.064)	

Adjusted for gender, age, dialysis vintage, body mass index, Age-adjusted Charlson Comorbidity Index, albumin, and C-reactive protein. LPA, light physical activity; MVPA, moderate-to-vigorous physical activity; ① Substituting walking; ② Substituting LPA; ③ Substituting MVPA; ④ Substituting daytime inactivity. ^*^p < 0.05; ^**^p < 0.01. Bold values in table indicate statistically significant results.

## 4 Discussion

This study examined the association between physical activity and sarcopenia (and its three parameters: skeletal muscle index, handgrip strength, and five-time sit-to-stand test) in peritoneal dialysis patients using the Isotemporal Substitution Model. The findings revealed that substituting 10 min of light physical activity for daytime inactivity significantly improved on the skeletal muscle index, handgrip strength, and five-time sit-to-stand test in physical activity patients. This indicates that even light intensity physical activities such as leisure activities and simple housework are associated with muscle mass, muscle strength and physical function in patients. It provides patients with more feasible exercise options, which helps enhance their interest in and participation in exercise. Additionally, when 10 min of walking was substituted for daytime inactivity, the time taken to complete five-time sit-to-stand test decreased significantly, and this effect was superior to that of light physical activity substitution. This indicates that walking is an effective form of exercise and is significantly associated with lower limb strength and balance ability in peritoneal dialysis patients. As a simple and accessible exercise, walking is suitable for most peritoneal dialysis patients. The results of this study offer new insights for exercise intervention in peritoneal dialysis patients; by optimizing daily activity schedules and increasing the proportion of light physical activity and walking is expected to improve muscle function, physical function and quality of life in patients.

### 4.1 Impact of physical activity level on sarcopenia incidence

This study found that walking, light physical activity, moderate-to-vigorous physical activity, and daytime inactivity had no correlation with the incidence of sarcopenia, and 10-min isotemporal substitution between different physical activity had no effect on the incidence of sarcopenia. These findings contrast with previous studies. A study conducted in Sweden (22) reported that moderate-to-vigorous physical activity was associated with a reduced incidence of sarcopenia. Similarly, Westbury et al. (20) found that each additional 1 h per day of moderate-to-vigorous physical activity reduced the risk of sarcopenia by 48%. In a Spanish elderly cohort, a significant reduction in the risk of sarcopenia was observed when moderate-to-vigorous physical activity was substituted for light physical activity (21), which is inconsistent with the results of this study. A possible reason for this discrepancy is that other studies enrolled elderly individuals, whereas this study focused on peritoneal dialysis patients, whose physical activity was dominated by light physical activity with minimal moderate-to-vigorous physical activity. Only 15.86% of the participants in our study engaged in moderate physical activity, and 0.93% engaged in vigorous physical activity. These low activity levels may have precluded demonstrating an effect of moderate-to-vigorous physical activity on sarcopenia.

Furthermore, peritoneal dialysis patients often experience a range of physical changes and physiological dysfunctions, such as electrolyte disorders, acid–base imbalance, and malnutrition (35). These conditions can further affect muscle health and function, complicating the association between physical activity and sarcopenia (36). In addition, the dialysis process itself may adversely effect on patients’ physical activity capacity and muscle function (25), potentially masking the association between physical activity level and sarcopenia.

### 4.2 Impact of physical activity level on skeletal muscle index

Isotemporal Substitution Model analysis of various types of physical activity showed that when 10 min of light physical activity was substituted for daytime inactivity, patients’ skeletal muscle index level increased significantly: an additional 10 min of light physical activity per day led to a 0.007 kg/m^2^ increase in skeletal muscle index. This finding is supported by other studies. A study by Pan et al. (37) found that total physical activity level and physical activity related to housework and leisure were negatively correlated with low skeletal muscle index or low handgrip strength, while sedentary behavior was positively correlated with low skeletal muscle index. Also, a Swedish study (23) found that isotemporal substitution of light physical activity for sedentary behavior was associated with increased skeletal muscle index levels. Therefore, peritoneal dialysis patients can use light physical activity to interrupt sedentary/recumbent behavior, which is beneficial for improving the level of skeletal muscle index.

When light physical activity replaces daytime inactivity, patients’ overall activity volume increases, which stimulates muscle protein synthesis, reduces muscle protein breakdown, and further promotes muscle growth, metabolism, and muscle mass improvement (38–40), thereby increasing skeletal muscle index levels. In addition, brisk walking can significantly increase the expression level of IGF-I in skeletal muscle (41), activate the proliferation of satellite cells, promote the differentiation of proliferated cells (42), improve skeletal muscle structure, repair damaged muscles, and indirectly enhance skeletal muscle index levels (41). Peritoneal dialysis patients presented a high prevalence of sedentary behavior. A 63% of the peritoneal dialysis patients were considered sedentary behavior (26). Sedentary behavior in peritoneal dialysis patients results in muscles remaining in a static state for an extended period, lacking necessary stimulation and exercise. Substituting daytime inactivity with light physical activity can reduce sedentary behavior, which may be associated with a lower risk of muscle degeneration and atrophy. Although current guidelines for peritoneal dialysis recommend moderate to vigorous physical activity rather than light physical activity, the findings of this study suggest that a significant improvement in the skeletal muscle index may be associated with a longer daily duration of consistent physical activity. This merits further in-depth research in the future.

### 4.3 Impact of physical activity level on handgrip strength

This study found when 10 min of light physical activity was substituted for daytime inactivity, patients’ handgrip strength increased significantly: each additional 10 min of light physical activity per day led to a 0.09 kg increase in handgrip strength. However, this finding is inconsistent with Sánchez’s study (21), in which he reported although isotemporal substitution of moderate-to-vigorous physical activity for sitting or light physical activity was associated with higher handgrip strength levels, isotemporal substitution of light physical activity for sedentary/recumbent behavior had no significant effect on handgrip strength. A possible reason for this discrepancy is that the subjects included in this study mainly engaged in light physical activity, with little participation in moderate-to-vigorous physical activity. Sustained light physical activity can exert a certain stimulatory effect on muscles, which helps promote the contraction and metabolism of muscle fibers, thereby maintaining the basic function of muscles. Substituting daytime inactivity with light physical activity can reduce sedentary/recumbent behavior and lower the risk of muscle atrophy, which in turn helps maintain and improve handgrip strength levels (41). In addition, as light physical activity is sustained, muscles gradually adapt to light physical activity physiologically, including thickening of muscle fibers and increased muscle protein synthesis, which contributes to enhancing muscle strength and endurance (39, 40). Therefore, substituting 10 min of daytime inactivity with light physical activity is significantly associated with increased handgrip strength in patients. Light physical activity should be encouraged to interrupt sedentary/recumbent behavior in peritoneal dialysis patients.

In this study, substituting daytime inactivity with light physical activity was significantly associated with increased handgrip strength, which reflects that light physical activity may exert a positive effect on muscle strength. This finding is aligned with a systematic review conducted by Ramsey et al. (43), who noted that older adults engaging in higher levels of total physical activity, light physical activity, moderate-to-vigorous physical activity, and lower levels of sitting demonstrated better upper arm strength. Moreover, compared with moderate-to-vigorous physical activity, light physical activity is generally more acceptable and feasible for peritoneal dialysis patients, which may improve adherence to their exercise.

### 4.4 Impact of physical activity level on five-time sit-to-stand test

This study also showed that when 10 min of walking time or light physical activity time was substituted for daytime inactivity, patients’ five-time sit-to-stand test time decreased significantly, and the reverse substitution also held true. Each additional 10 min of walking per day shortened five-time sit-to-stand test time by 0.077 s; each additional 1 h of light physical activity per day shortened five-time sit-to-stand test time by 0.045 s. A multicenter study (44) reported similar findings that walking and resistance exercise can improve lower limb muscle strength and shorten five-time sit-to-stand test time. When walking time replaced daytime inactivity, patients’ lower limb muscles received more exercise, which helped improve the strength and endurance of lower limb muscles, thereby shortening five-time sit-to-stand test time. When light physical activity time replaced daytime inactivity, five-time sit-to-stand test time was also shortened. This is consistent with the results of a Japanese study (45), which found that substituting light physical activity for sedentary behavior was associated with a decrease in five-time sit-to-stand test time. Light physical activity can provide beneficial stimulation and exercise for the lower limb muscles. In this study, light physical activity primarily consisted of walking, which explains its observed effect. Therefore, based on the results, it is recommended that peritoneal dialysis patients can reduce the time taken for the five-time sit-to-stand test through walking and light physical activity. Walking and light physical activity should be encouraged to interrupt sedentary/recumbent behavior in peritoneal dialysis patients.

## 5 Limitations

However, this study has several limitations. First, it adopted a cross-sectional study design, and the results are derived from the collected data. It limits the ability to infer a causal relationship between physical activity and peritoneal dialysis-related sarcopenia. Second, physical activity levels were assessed using a questionnaire survey, which may lead to recall bias. Third, due to the small number of patients participating in moderate-to-vigorous physical activity in this study, no effect of substituting moderate-to-vigorous physical activity for sedentary/recumbent behavior on the three sarcopenia parameters was observed. Finally, regarding the assessment of physical function, the sole use of the 5-STS test in this study has certain limitations. We did not adopt multiple measurement methods such as the SPPB and walking speed to evaluate physical function. Future studies should adopt longitudinal designs and utilize wearable devices to objectively monitor physical activity levels in peritoneal dialysis patients, thereby clarifying the causal relationship between physical activity and sarcopenia. Furthermore, rigorous clinical trials are needed to address unanswered questions regarding the cumulative effect of physical activity and the minimum intervention duration required to reverse or delay the progression of sarcopenia and its key parameters (skeletal muscle index, handgrip strength, five-time sit-to-stand test).

## 6 Conclusion

This study found that substituting daytime inactivity with light physical activity was significantly associated with the parameters (skeletal muscle index, handgrip strength, the five-time sit-to-stand test) of sarcopenia. Substituting daytime inactivity with walking was significantly associated with the five-time sit-to-stand test. When formulating exercise plans, not only the amount of exercise but also the exercise type and intensity acceptable to patients should be considered to improve exercise compliance. It is recommended that peritoneal dialysis patients should prioritize walking and light physical activity. For peritoneal dialysis patients with sedentary or recumbent behavior, it is recommended to substitute sedentary behavior with walking, light physical activity. An interruption strategy for sedentary/recumbent behavior (e.g., inserting 10 min of walking or light physical activity after every 30 min of sedentary/recumbent behavior) should be implemented to improve skeletal muscle mass, muscle strength, and physical function.

## Data Availability

The original contributions presented in the study are included in the article/Supplementary material, further inquiries can be directed to the corresponding author.
