# Supplementary material for: Association of different types of physical activity with sarcopenia and its parameters in peritoneal dialysis patients: an isotemporal substitution analysis
Source: Front Nutr. 2026 Feb 25;13:1780207. doi: 10.3389/fnut.2026.1780207 (PMC12975755; doi:10.3389/fnut.2026.1780207)
Supplement: Supplementary file 1 [file Supplementary_file_1.docx]

**Supplementary table S1 Basic Characteristics of the Study Subjects**

|  | | PD patients(n=643) | Non-sarcopenia（n=562） | sarcopenia（n=81） | *t/x^2^/z* | *p* |
| --- | --- | --- | --- | --- | --- | --- |
| Age [year,*$\bar{x}$*± s] |  | 48.04 ± 12.77 | 47.29 ± 12.05 | 53.27 ± 16.06 | **-4.00**^①^ | **＜0.01** |
| Sex [n (%)] | Male | 326 (50.70) | 281 (50.00) | 46 (56.79) | 1.31^②^ | 0.25 |
|  | Female | 317 (49.30) | 281 (50.00) | 35 (43.21) |  |  |
| SMI [kg/m^2^,*$\bar{x}$*± s] |  | 6.90 ± 1.16 | 7.06 ± 1.12 | 5.81 ± 0.78 | **9.70**^①^ | **＜0.01** |
| HGS [kg,*$\bar{x}$*± s] |  | 27.46 ± 8.60 | 28.47 ± 8.49 | 20.49 ± 5.66 | **8.19**^①^ | **＜0.01** |
| 5-STS [s,M(P_25_,P_75_)] |  | 8.56 (7.09,10.17) | 8.50 (6.94,10.00) | 10.00 (8.22,13.70) | **-5.80**^③^ | **＜0.01** |
| Transport walking [n (%)] | Present | 86 (13.37) | 79 (14.06) | 7 (8.64) | 1.79^②^ | 0.18 |
|  | Absent | 557 (86.63) | 483 (85.94) | 74 (91.36) |  |  |
| Fitness walking [n (%)] | Present | 77 (11.98) | 73 (12.99) | 4 (4.94) | **4.35**^②^ | **0.04** |
|  | Absent | 566 (88.02) | 489 (87.01) | 77 (95.06) |  |  |
| Leisure walking [n (%)] | Present | 437 (67.96) | 378 (67.26) | 59 (72.84) | 1.01^②^ | 0.31 |
|  | Absent | 206 (32.04) | 184 (32.74) | 22 (27.16) |  |  |
| Total walking [n (%)] | Present | 521(81.03) | 456 (81.14) | 66 (81.48) | 0.01^②^ | 0.91 |
|  | Absent | 122 (18.97) | 106 (18.86) | 15 (18.52) |  |  |
| LPA [n (%)] | Present | 448 (69.67) | 388 (69.04) | 60 (74.07) | 0.85^②^ | 0.36 |
|  | Absent | 195 (30.33) | 174 (30.96) | 21 (25.93) |  |  |
| MPA [n (%)] | Present | 102 (15.86) | 91 (16.19) | 11 (13.58) | 0.36^②^ | 0.55 |
|  | Absent | 541 (84.14) | 471 (83.81) | 70 (86.42) |  |  |
| VPA [n (%)] | Present | 6 (0.93) | 6 (1.07) | 0 (0) | - | - |
|  | Absent | 637 (99.07) | 556 (98.93) | 81 (100.00) |  |  |
| Total PA consumption [MET-h/w, M(P_25_,P_75_)] |  | 24.50 (10.50,47.25) | 26.13(10.50,49.00) | 20.50 (10.50,42.00) | -1.10^③^ | 0.27 |
| Dialysis vintage [month, M(P_25_,P_75_)] |  | 23.00 (7.00,50.00) | 22.00 (7.00,45.00) | 35.00 (15.00,72.50) | **-3.21**^③^ | **＜0.01** |
| BMI [kg/m^2^,*$\bar{x}$*± s] |  | 21.91 ± 3.17 | 22.28 ± 3.12 | 19.32 ± 2.17 | **8.27**^①^ | **＜0.01** |
| ACCI [score,*$\bar{x}$*± s] |  | 3.03 ± 1.35 | 2.94 ± 1.29 | 3.63 ± 1.60 | **-4.35**^①^ | **＜0.01** |
| Albumin [g/L,*$\bar{x}$*± s] |  | 38.73 ± 4.61 | 39.13 ± 4.53 | 38.71 ± 4.55 | 0.64^①^ | 0.52 |
| C-reactive protein [mg/L,M(P_25_,P_75_)] |  | 1.48 (0.61,4.35) | 1.49 (0.61,4.40) | 1.36 (0.62,3.90) | -0.24^③^ | 0.81 |

Note: ^①^Independent samples t-test; ^②^Chi-square test; ^③^Mann-Whitney U test.

SMI: skeletal muscle index; HGS: handgrip strength; 5-STS: five-time sit-to-stand test; LPA: light physical activity；MPA: moderate physical activity; VPA: vigorous physical activity; PA: physical activity; BMI: body mass index; ACCI: Age-adjusted Charlson Comorbidity Index.

**Supplementary table S2 Single and Partitioned Models of Physical Activity Levels and Sarcopenia Incidence**

| Category | Walking | | LPA | | MVPA | | Daytime inactive time | |
| --- | --- | --- | --- | --- | --- | --- | --- | --- |
|  | *β* | *OR* (95%CI) | *β* | *OR* (95%CI) | *β* | *OR* (95%CI) | *β* | *OR* (95%CI) |
| Model1 | -0.061 | 0.941  (0.868, 1.020) | -0.015 | 0.985  (0.950, 1.022) | -0.060 | 0.942  (0.835, 1.063) | 0.007 | 1.007  (0.990, 1.024) |
| Model2 | -0.061 | 0.941  (0.867, 1.022) | -0.015 | 0.985  (0.949, 1.023) | -0.058 | 0.944  (0.836, 1.066) | 0.002 | 1.002  (0.984, 1.020) |

Note: LPA：light physical activity；MVPA：moderate-to-vigorous physical activity. Model 1 (single-model) was adjusted for gender, age, dialysis vintage, body mass index, Age-adjusted Charlson Comorbidity Index, albumin, and C-reactive protein. Model 2 (partition-model) = (*β*1)Walking + (*β*2)LPA + (*β*3)MVPA + (*β*4)Daytime Inactive Time + (*β*6)Covariates, it was adjusted for gender and age on the basis of Model 1. The regression coefficient corresponds to the effect of a 10-minute increase in each activity. ^*^*P* < 0.05; ^**^*P* < 0.01.

**Supplementary table S3 Single and Partitioned Models of Physical Activity Levels and Skeletal Muscle Index**

| Category | Walking | LPA | MVPA | Daytime Inactive Time |
| --- | --- | --- | --- | --- |
|  | *β* (95%CI) | *β* (95%CI) | *β* (95%CI) | *β* (95%CI) |
| Model 1 | 0.010  (-0.002, 0.021) | 0.005  (-0.001, 0.011) | 0.008  (-0.008, 0.024) | -0.003  (-0.006, 0.001) |
| Model 2 | 0.009  (-0.003, 0.020) | 0.005  (-0.002, 0.011) | 0.007  (-0.009, 0.023) | -0.002  (-0.005, 0.001) |

Note: LPA：light physical activity；MVPA：moderate-to-vigorous physical activity. Model 1 (single-model) was adjusted for gender, age, dialysis vintage, body mass index, Age-adjusted Charlson Comorbidity Index; albumin, and C-reactive protein; Model 2 (partition-model) = (*β*1)Walking + (*β*2)LPA + (*β*3)MVPA + (*β*4)Daytime Inactive Time + (*β*6)Covariates, it was adjusted for gender, age, dialysis vintage, body mass index, Age-adjusted Charlson Comorbidity Index, albumin, and C-reactive protein on the basis of Model 1. The regression coefficient corresponds to the effect of a 10-minute increase in each activity. ^*^*P* < 0.05; ^**^*P* < 0.01.

**Supplementary table S4 Single and Partitioned Models of Physical Activity Levels and Handgrip Strength**

| Category | Walking | LPA | MVPA | Daytime Inactive Time |
| --- | --- | --- | --- | --- |
|  | *β*(95%CI) | *β*(95%CI) | *β*(95%CI) | *β*(95%CI) |
| Model 1 | 0.064  （-0.039, 0.167） | **0.089^**^**  （0.033, 0.145） | 0.006  （-0.140, 0.153） | -0.012  （-0.042, 0.017） |
| Model 2 | 0.074  （-0.030, 0.178） | **0.092^**^**  （0.034, 0.151） | 0.008  （-0.139, 0.155） | 0.002  （-0.029, 0.033） |

Note: LPA：light physical activity；MVPA：moderate-to-vigorous physical activity. Model 1 ( single-model) was adjusted for gender, age, dialysis vintage, body mass indexI, Age-adjusted Charlson Comorbidity Index, albumin, and C-reactive protein. Model 2 (partition-model) = (*β*1) Walking + (*β*2) LPA + (*β*3) MVPA + (*β*4) Daytime Inactive Time + (*β*6) Covariates; it was adjusted for gender, age, dialysis vintage, body mass index, Age-adjusted Charlson Comorbidity Index, albumin, and C-reactive protein on the basis of Model 1. The regression coefficient corresponds to the effect of a 10-minute increase in each activity. ^*^*P* < 0.05; ^**^*P* < 0.01.

**Supplementary table S5 Single and Partitioned Models of Physical Activity Levels and Five-Time Sit-to-Stand Test**

| Category | Walking | LPA | MVPA | Daytime Inactive Time |
| --- | --- | --- | --- | --- |
|  | *β*(95%CI) | *β*(95%CI) | *β*(95%CI) | *β*(95%CI) |
| Model 1 | **-0.066^*^**  （-0.124, -0.007） | -0.031  （-0.063, 0.001） | -0.011  （-0.094，0.072） | **0.027^**^**  （0.010, 0.043） |
| Model 2 | -0.056  （-0.115, 0.003） | -0.023  （-0.056, 0.010） | 0.003  （-0.080，0.086） | **0.022^*^**  （0.004, 0.039） |

Note: LPA：light physical activity；MVPA：moderate-to-vigorous physical activity. Model 1 (single-model) was adjusted for gender, age, dialysis vintage, body mass index, Age-adjusted Charlson Comorbidity Index, albumin, and C-reactive protein. Model 2 (partition-model) = (*β*1) Walking + (*β*2) LPA + (*β*3) MVPA + (*β*4) Daytime Inactive Time + (*β*6) Covariates; it was adjusted for gender, age, dialysis vintage, body mass index, Age-adjusted Charlson Comorbidity Index, albumin, and C-reactive protein on the basis of Model 1. The regression coefficient corresponds to the effect of a 10-minute increase in each activity. ^*^*P* < 0.05; ^**^*P* < 0.01.
